# The effect of courage on stress: The mediating mechanism of behavioral inhibition and behavioral activation in high-risk occupations

**DOI:** 10.3389/fpsyg.2022.961387

**Published:** 2022-08-18

**Authors:** Jia Wang, Dingyu Sun, Juan Jiang, Huizhong Wang, Xiaotong Cheng, Qianying Ruan, Yichao Wang

**Affiliations:** ^1^Department of Developmental Psychology of Armyman, School of Psychology, Army Medical University, Chongqing, China; ^2^College of Basic Medicine, Army Medical University, Chongqing, China; ^3^School of Psychology, Army Medical University, Chongqing, China; ^4^Unit 32298 of the Chinese People’s Liberation Army, Weifang, China; ^5^Department of Blood Transfusion, The First Affiliated Hospital of Army Medical University, Chongqing, China; ^6^Graduate School, Army Medical University, Chongqing, China

**Keywords:** courage, stress, high-risk occupations, behavioral inhibition, behavioral activation

## Abstract

Employees in high-risk occupations are exposed to tremendous work acute stress or prolonged stress disorders that are likely to undermine the health and organizational effectiveness. Based on positive psychology, courage which refers to behavioral approach despite the experience of fear could buffer the negative effects on stress. However, there is little known about the mechanisms by which courage decreases the risk of stress. Motivational systems may play an underlying role in this process, as behavioral inhibition system (BIS) is inhibited and behavioral activation system (BAS) is evoked by risk or threat. The current study aimed to examine the mediating effects of behavioral inhibition and activation on the relationship between courage and stress in the high-risk occupations. This study recruited 1,761 high-risk employees aged from 18 to 27 (*M* = 19.32; *SD* = 4.14) with a cluster sampling method who completed Courage Measure (CM), the BIS/BAS Scales and the Psychological Stress Evaluation Test (PSET). The correlation and mediation analyses examined the inter-variable correlations as well as the underlying mechanism between courage and stress. The results support the hypothesis and reveal that the behavioral inhibition mediates the association between courage and stress (*B*_*indirect*_ = −0.02, *p* < 0. 01, 95%CI = −0.03 to −0.003). The behavioral activation of fun seeking mediates the association between courage and stress as well (*B*_*indirect*_ = −0.04, *p* < 0. 01, 95%CI = −0.058 to −0.029). These findings suggest that behavioral inhibition and activation of fun seeking play imperative mechanism underpinning the buffering effect of courage on stress. Other theoretical and applied implications for desensitizing stress in the high-risk occupations are discussed.

## Introduction

High-risk occupations (e.g., firefighters, policemen, soldiers, etc.) refer to “non-fatal injury and illness rate that was two more times than the national rate per 10,000 full time-equivalent workers” (Siordia and Galley, 2020). Those who committed to duties are usually accustomed to danger or threat of occupational surroundings that expose the high-risk employee on overwhelming stress conditions (Lee et al., 2020), in which undermine employees’ health (Stevelink et al., 2018; Jonker et al., 2020) and organizational effectiveness (Tucker et al., 2005; Vinokur et al., 2011). The deteriorated affects have shown on the short and long approaches. For the short-term responses, acute threats (e.g., fire rescue, arrest counter-terrorism operation, etc.) evoked individuals’ emergency reactions to cope with stress. This fight or flight response arose from biologically stress center of hypothalamus activating the autonomic nervous system and hypothalamus-pituitary-adrenal (HPA) (Glavin, 1985). This physiological changes caused by acute stress might take a toll on health because of stress-induced immune dysfunction (Glaser and Kiecolt-Glaser, 2005; Wolkow et al., 2015) and have a deterioration effect on job performance due to impairing working memory (Xin et al., 2020). For the long-term responses, based on the general adaptation syndrome model, there are three stages during stress reaction (i.e., alarm, resistance, and exhaustion, respectively). Following exposure to stressors, alarm is the first periods of bodily arousal that prepares the organism for vigorous and adaptive coping. If stressors are prolonged, the body enters the resistance stage in which organism continues to endure further cumulative effects of prolonged stressors. However, if the stressors are sufficiently lasting or irreversible, the organism chronically enters into exhaustion and individuals undergo mental disorders, illness, even death (Selye, 1976; O’Connor et al., 2021). Additionally, plentiful studies have suggested that individuals in the high-risk occupations are exposed to tremendous stress which increases the risk of stress disorders. For example, Andersen investigated 2,678 employees who were exposed to work-related stress in high-risk occupations. The results showed that 13.9% of employees reported short-term post-traumatic stress disorder (PTSD), and 17.9% reported long-term PTSD (Andersen et al., 2019). Wagner et al. (2021) have systematically reviewed that the prevalence of PTSD varied widely from 0.90 to 32.5% (*M* = 12.3%) for firefighters of which was significantly higher compared with rates in the normal population (8%), and the severity of stressors exposure bears a stronger relationship to the development of PTSD. As for the police following routine work-related critical incident exposure, the PTSD prevalence varied considerably across studies from 0 to 44% (*M* = 14.87%) (Wagner et al., 2020). Moreover, a study on military cohort enrolled 8,093 personnel who were involved in the wars in Iraq and Afghanistan, highlighting an increasing prevalence (6.2%) for PTSD (Stevelink et al., 2018). Consequently, in risky workplaces, employees experience an increasing risk of stress sequelae, which in turn predicts subsequent deterioration in mental health, wellbeing and may lead to a decline in organizational effectiveness. It is of great practical significance to explore the protective mechanism on overwhelming stress, especially in the high-risk occupations, in which the incidence rates of stress related disorders and symptoms are dramatically higher than those in other contexts.

### The relationship between courage and stress

Courage, defined as behavioral approach despite the experience of fear (Norton and Weiss, 2009), might provide enough inner strength to buffer the stress in high-risk circumstances deriving from positive psychology (Peterson and Seligman, 2004). Based on the subjective experience model, courage is the physiological and psychological response of fear induced by the awareness of risk, and an actor’s performance in the face of fear to achieve a designated purpose (Hannah et al., 2007). Previous studies extracting the main components of courage were risk (Petersen, 2004; Christopher, 2007; Woodard and Pury, 2007), fear (Putman, 2001; Woodard, 2001; Pears, 2004) and noble/good act (Pury and Starkey, 2009; Rate, 2009). From the perspective of evolution, appropriate fear has an adaptive function for individuals’ survival when they are confronted with threat by enhancing individuals’ vigilance and coping readiness. Once the intense horror is evoked, employees might lie in freeze, or even disability to duty (Ralphs, 2013). For instance, above 60% soldiers in the combat are unable to complete the mission because of overpowering terror (Daddis, 2007; Gondra et al., 2009). However, courageous soldiers are capable of controlling and enduring fear to achieve the goals (Dollard and Horton, 1944). Howard and Reiley (2020) study enrolled 368 cadets from the United States Air Force Academy. The study showed that courage was significantly related to military performance (β = 0.13, *p* < 0.05). In addition, Rachman’s research clearly showed that among bomb-disposal operators, parachutists and veterans, a higher level of courage linked with declining stress led to an optimal level of operational performance (Rachman, 1982, 2004). Further, positive psychologists have linked courage to wellbeing, life satisfaction and the alleviation of depression and distress (Gayton and Kehoe, 2019). According to a survey of 1,000 soldiers in brigade units in the Iraq War, as a protective factor, courage could effectively buffer the damaging effects of combat stress on mental health. The results showed that brave soldiers who were able to effectively handle or regulate fear showed less combat fatigue, anger, post-traumatic stress responses, but much more positive deployment experience (McGurk and Castro, 2009). Similarly, Neria et al. (2000) found that the incidence of PTSD in heroes who were decorated medals for bravery in combat was significantly lower than that of non-decorated bravery soldiers after 25 years.

### The mediating role of behavioral inhibition system/behavioral activation system in the relationship between courage and stress

In brief, there is substantial evidence that courage has a negative association with stress. Nonetheless, the underlying mechanism processes remain unclear. One plausible path might take the motivational systems into consideration. There are two independently motivational systems in human beings: BIS and BAS. Gray’s neuropsychological reinforcement sensitivity theory (Gray and McNaughton, 2000) postulated that BIS was sensitive to stimuli of punishment or non-ward and inhibit the action to avoid potentially negative consequences; BAS was sensitive to stimuli of reward or the termination of punishment and activate action to approach the potentially positive consequences. On the one hand, ample studies have linked the courage with motivational systems that individuals with high levels of courage may show greater inhibition of the BIS system and proneness of BAS system. Boldness is considered a disposition theorized to be rooted in low biological threat sensitivity (Patrick et al., 2009, 2019), in which correlate negatively with fear (Rachman, 1982; McMillan and Rachman, 1988; Brislin et al., 2017), pain (McCabe et al., 2015) and other defensive behaviors (Dindo and Fowles, 2011), positively with heroism involved attaining the prosocial goal or sacrifice beyond duty (Franco et al., 2011; Goethals and Allison, 2012). On the other hand, the available literature suggests that an imbalance between BIS and BAS activation across multiple employees could deteriorate mood and elicit simultaneous physiological and psychological stress responses. For example, a greater activation of inhibitory behaviors will produce larger negative affect; a stronger approach to appetitive stimuli produces more positive affects (Merchán-Clavellino et al., 2019). Moreover, a meta-analysis systematically reviewing 204 studies found that BIS could significantly predict depression (β = 0.37) and anxiety (β = 0.35), while BAS only significantly predicting depression (β = −0.07) (Benjamin et al., 2020). Hinnant et al. (2017) indicated that BAS was positively related to substance use for individuals with high-level stress. However, under the stress, it generally leads to the enhanced avoidance, and induces approach in individuals prone to aggression (Vogel and Schwabe, 2019). Contractor et al. (2013) using confirmatory factor analysis and chi-square tests demonstrated a significantly greater association with BIS severity compared to BAS severity for PTSD. The results confirmed the 194 patients suffered from both chronic pain and posttraumatic stress symptoms (Serrano-Ibáñez et al., 2019).

Thus, to protect employees in high-risk occupations stemming from overwhelming stress, the possible mechanisms need to be investigated. Extant studies have illuminated that BIS/BAS both related with courage and stress; nevertheless, little research has revealed the mechanism on the effect of courage on stress. The identification of the underlying mechanisms are indispensable requirements for developing interventions based on courage for promoting high-risk employees’ health and organizational effectiveness. Thus, our hypotheses are behavioral inhibition and activation that mediate the association between courage and stress. To be specifically, we posit that courage can alleviate stress by inhibiting the BIS, whereas activating the BAS.

## Materials and methods

### Participants

There were 1,761 participants recruited from high-risk occupations of firefighters voluntarily and anonymously with cluster sampling. Participants had a mean age of 19.32 ± 4.14 years, range from 18 to 27 years; all of them were male. A total of 396 had a low education level (22.49%); 1,087 had a moderate education level (61.72%); 278 had a moderate education (15.79%). The study was approved by the ethics committee of Army Medical University (No. 2020-024-03).

### Measures

#### Courage

Courage was assessed by the Courage Measure (CM) (Norton and Weiss, 2009). The CM was developed by Norton and his colleges and provided an operational definition of courage as persistence or perseverance despite experiencing fear in high-risk situations. The scale consists of 12 items rated from 1 (never) to 7 (always). The CM score is the sum of the scores for each item; the Cronbach’s α coefficient was 0.92. The test-retest correlation at 3 weeks was 0.66 (*p* < 0.001). In our sample, the higher the sum of score, the higher individuals’ bravery level was possessed. The majority of researchers agree with this measurement of the CM (47–50). In the present study, Cronbach’s α coefficient was 0.81, and the model fit indices were perfect (CMIN/DF = 11.55, NFI = 0.91, IFI = 0.92, CFI = 0.92, RMSEA = 0.07), indicating that reliability and validity were good.

#### Behavioral inhibition and activation

Behavioral inhibition and activation were assessed with the Chinese version of the BIS/BAS Scales (BIS/BAS) developed by Carver and White (Charles and Teri, 1994), including one behavioral inhibition subscale and three behavioral activation subscale of reward responsiveness, drive and fun seeking. The BIS/BAS consist of 20 items rated from 1 (strong agreement) to 4 (strong disagreement); the BIS/BAS scores are the sum from subscales’ items. In our sample, the data was reversed scoring in each item, so that the more scores imply being greater in the BIS/BAS traits. In the Chinese version of the BIS/BAS (Li et al., 2008), confirmatory factor analysis indicated that there were four factors involved as same as the original version, and the model fit indices were perfect (CMIN/DF = 1.52, NFI = 0.92, IFI = 0.89, CFI = 0.89, RMSEA = 0.04); the test-retest correlation at 2 months were 0.59 0.69 (*p* < 0.01). In the present study, Cronbach’s α coefficient was 0.81, and model fit indices were perfect (CMIN/DF = 8.54, NFI = 0.87, IFI = 0.88, CFI = 0.88, RMSEA = 0.06), indicating that reliability and validity were good.

#### Stress

Stress was assessed with the Psychological Stress Evaluation Test (PSET) consisting of 10 items (Chao et al., 2003) rated from 1 (none) to 3 (usually). The PSET score is the sum score from subscales’ items (e.g., I feel tense, irritable and anxious; I feel mentally and physically tired.). In our sample, the higher the sum of score, the higher individuals’ stress level was possessed. The coefficients of 10 items in PSET with general score were above 0.3 (0.39 ∼ 0.56, *p* < 0.01); the Cronbach’s α coefficient was 0.76; the test-retest correlation at 2 weeks was 0.33 ∼ 0.60 (*p* < 0.01); the coefficients of PSET total score with factors of SCL-90 were 0.48 ∼ 0. 67 (*p* < 0.01). In the present study, Cronbach’s α coefficient was 0.81, and model fit indices were perfect (CMIN/DF = 10.21, NFI = 0.93, IFI = 0.93, CFI = 0.93, RMSEA = 0.07), indicating that reliability and validity were good.

### Procedure

The survey was guided by a professional psychology researcher with standard procedure. Only participants who completed the whole questionnaire were included. Questionnaires with missing or irregular answers were excluded.

### Statistical analysis

The correlation analyses and testing common method bias of the outcome measures were performed by using SPSS 21.0. Pearson bivariate correlations were calculated to identify inter-variable correlations. The Harman’s single-factor test was performed to test the common method bias on self-reported questionnaires. The direct model was verified and mediation model was tested with structural equation modeling by using AMOS 24.0 in the bootstrap method with 5,000 times of deviation correction.

## Results

### Testing common method biases

Since all the data in this study were self-reported by participants, Harman single-factor test was adopted to test common method bias. The exploratory factor analysis of all the three variables shows that the variance explanation rate of first factor is only 17.79%, less than the critical standard of 40%; 8 factors with eigenvalues greater than 1 are analyzed. Therefore, the results showed that there was no serious common method bias in the data.

### Correlations among courage, behavioral inhibition and activation and stress

A simple correlation analysis revealed that courage was negatively associated with stress (*r* = −0.41, *p* < 0. 01), behavioral inhibition (*r* = −0.10, *p* < 0. 01), fun seeking (*r* = −0.17, *p* < 0. 01), whereas positively associated with drive (*r* = 0.20, *p* < 0. 01), reward responsiveness (*r* = 0.16, *p*< 0. 01). Other inter-variable correlations were in Table 1.

**TABLE 1:** The correlations for all variables.

	1	2	3	4	5	6
1. Courage	1					
2. Stress	−0.41**	1				
3. Behavioral inhibition	−0.10**	0.19**	1			
4. Drive	0.20**	−0.01	0.35**	1		
5. Reward responsiveness	0.16**	−0.02	0.49**	0.55**	1	
6. Fun seeking	−0.17**	0.26**	0.43**	0.44**	0.43**	1

**p < 0.01.

### Direct effect of courage on stress

**FIGURE 1:**
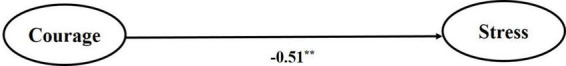
Direct effect of courage on stress. **p< 0.01.

In the direct effect model of courage on stress, courage was entered as independent variables; stress was entered as the dependent variable. Figure 1 showed that the direct effect on courage to stress is −0.51, *p* < 0. 01. The results of structural equation modeling showed that the overall model yielded a satisfactory fit (CMIN/DF = 6.06, NFI = 0.99, IFI = 0.99, CFI = 0.99, RMSEA = 0.05).

### Mediating mechanism of behavioral inhibition

**FIGURE 2:**
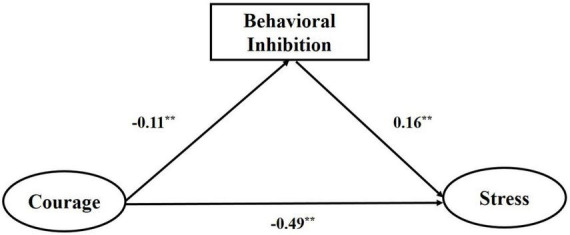
Behavioral inhibition as a mediator through which courage decrease stress. **p < 0.01.

In the mediation analysis of behavioral inhibition, courage was entered as independent variables; stress was entered as the dependent variable and behavioral inhibition as the mediator (Figure 2). Table 2 showed that the relationship between courage and stress was partially mediated by behavioral inhibition (*B*_*indirect*_ = −0.02, *p* < 0. 01, 95%CI = −0.03 to −0.003). The results of structural equation modeling showed that the overall model yielded a satisfactory fit (CMIN/DF = 3.90, NFI = 0.92, IFI = 0.99, CFI = 0.99, RMSEA = 0.04).

**TABLE 2:** Test the mediation model of behavioral inhibition.

IV	DV		Coeff.	SE	95%CI	
					LL	UL
Courage	Stress	Total effect	−0.51	0.026		
		Direct effect	−0.49	0.028		
		Indirect effect	−0.02	0.007	−0.030	−0.003

IV, independent variable; DV, dependent variable; CI, confidence interval; LL, lower level; UL, upper level.

### Mediating mechanism of fun seeking

**FIGURE 3:**
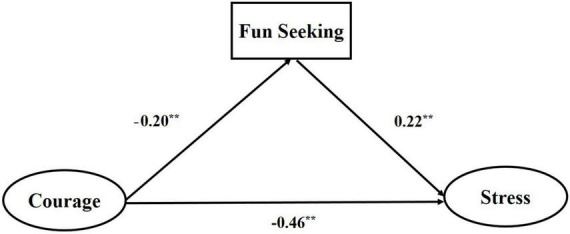
Fun seeking as a mediator through which courage decrease stress. ^**^p < 0.01.

Drive and reward responsiveness were not significantly correlated with stress (*p* > 0.05), hence these two mediators were excluded from mediation analysis (Shrout and Bolger, 2002). In the mediation analysis of behavioral activation, courage was entered as independent variables, whereas stress as the dependent variable, and fun seeking as the mediator. In mediation of fun seeking (see Figure 3 and Table 3), the mediating effect was significant (*B*_*indirect*_ = −0.04, *p* < 0. 01, 95%CI = −0.058 to −0.029) and the overall model yielded a satisfactory fit (CMIN/DF = 11.31, NFI = 0.96, IFI = 0.97, CFI = 0.97, RMSEA = 0.07).

**TABLE 3:** Test the mediation model of fun seeking.

IV	DV		Coeff.	SE	95%CI	
					LL	UL
Courage	Stress	Total effect	−0.51	0.027		
		Direct effect	−0.47	0.028		
		Indirect effect	−0.04	0.009	−0.058	−0.029

IV, independent variable; DV, dependent variable; CI, confidence interval; LL, lower level; UL, upper level.

## Discussion

The present study evaluated the hypothesis that BIS/BAS mediate the relationship between courage and stress. Before a mediation model could be assessed, the inter-correlations of CM, BIS/BAS, and PSET had to be analyzed. First, BIS was negatively related to courage, indicating that high levels of BIS was to some extent associated with lower levels of courage. This result was in keeping with the idea that courage was equivalently considered with the inclination of persistence despite fear (PDF) (Howard and Murry, 2020), in which fear emotion positively induced the BIS and vice versa. Compassed the fear, the BIS might be inhibited (Demaree et al., 2009; Ralphs, 2013). Further, BIS was positively related to stress for the reason that bulk of studies demonstrated that BIS consistently predicted negative affect including stress (Carver, 2004; Bijttebier et al., 2009). Second, the correlations between BAS-drive, reward responsiveness, fun seeking and courage were complicated. There were positive association between the courage and BAS-drive, reward responsiveness as predicted since the more brave employees were more likely to persist in pursuing toward noble goals (Taubitz et al., 2015; Costumero et al., 2016). However, fun seeking was negatively associated with courage, which was inconsistent with previous studies that there was a strong positive correlation between sensation seeking and courage (Muris, 2009). One possible reason was that fun seeking is more related to impulsivity during which individuals might need to inhibit the impulsivity toward the noble goals since manifesting the inversely motivational direction (Caseras et al., 2003; Smillie et al., 2006). According to Moeller et al. (2001) considering impulsivity as a predisposition toward rapid, unplanned reactions to internal or external stimuli disregarded the negative consequences of these reactions to the impulsive individual or to others, with high comorbidity with psychiatric disorders. For example, impulsivity would significantly predict the psychological distress (*B*_*direct*_ = −0.5) for the impairment of cognitive control (McKewen et al., 2019) and make adaptive decisions (Hogarth et al., 2012). Consequently, individuals with high-level courage might experience lower-level fun seeking (impulsivity) for driving goals with enough cognitive resource to buffering stress. In addition, the correlation between drive, reward responsiveness and stress was clearly non-significant. These results indicated that BAS-drive, reward responsiveness were sensitive to positive stimulus and evoked positive affect, such as happiness (Charles and Teri, 1994). On the contrary, fun seeking to some extent related with impulsivity. It might indirectly have a link with stress. For example, Moustafa et al. (2017) found the impulsivity was associated with stress.

Courage is an ancient predisposition that has played a protective role on the stress. Consistent with previous studies (Maddi, 2008; McGurk and Castro, 2009; Howard and Reiley, 2020), our study found that the enhanced level of courage would predict the decreasing stress level since the direct effect of courage on stress was significant (*B*_*direct*_ = −0.51). Specifically, one possibility on buffering stress is that courage might positively linked with a self-directed coping (i.e., having a positive attitude to problem-solving) whereas negatively predicted with self-avoidant coping (i.e., using avoidance strategies and having a transcendent orientation). It acts on coping by activating psychological resources that can help actors to sustain and persevere, which can manage effectively and surmount the damage to cognitive function under conditions of stress (Magnano et al., 2017; Abdollahi et al., 2022). Besides, hardiness, as a pattern of courage and strategy that facilitates turning stressful circumstances from potential threats into growth opportunities by developing 3C’s (i.e., challenge, commitment, control) hardy coping, social support and self-care (Maddi, 2008). Another possibility alleviating stress is that the BIS/BAS was elicited as a previous hypothesis that it mediates the relationship between courage and stress.

For BIS, our results align with hypothesis that courage would effectively reduce stress by inhibiting the BIS, consistent with a previous study that those who with high boldness were associated with greater accuracy and efficient of switching task responding under external threat as compared with safe blocks. This finding suggests that high boldness may act as a buffer against the impairing effects of external threat, specifically in terms of guiding behavior effectively and accurately in the service of goal attainment within high-stress environments (Yancey et al., 2019). Employees could be confronted with tremendous threats or stressors due to their risky environments, such as physical injuries or death (Mitcheli et al., 2013; Rajabi et al., 2020). When human beings encounter risks, the BIS system is activated engaging in defensive behaviors, by which these freeze, flight and avoidance behaviors can enhance people’s chances of survival in life-endangering situations (Rösler and Gamer, 2019). Defensive behaviors, such as evolutionarily adaptive responses, can help people avoid the potential danger (Blanchard, 2017). However, in dangerous occupations, employees must endure or overcome risks to complete their duties or missions. In order to do that, they need to conquer fear, and achieve the noble goal in which might mean to inhibit the BIS system. On the basis of the battlemind model (McGurk and Castro, 2009) and the cognitive theory (Woodard, 2001) of courage, threat-adverse emotional response process was embedded with risks when accompanied by negative emotions (e.g., fear, anger and sadness, etc.), which in turn automatically evoked BIS to protect them from danger. After that, individuals calculated the meaningfulness of the noble goal *via* cost/benefit calculation. If the reward exceeds the cost, it means the duty enjoys high meaningfulness. The courageous actions stimulated by enduring and persevering the adversity might through inhibit the BIS to achieve the goal-oriented duties or missions. The courageous behaviors further inhibits the BIS by activating persistence and endurance of suffering to accomplish goal-oriented duties or missions, in which BIS both responsible for the processing of aversive stimuli and goal conflicts (Espinoza Oyarce et al., 2021). For example, the more valorous people are, the larger fear they endure, whereas the less behavioral avoidance they experienced (Norton and Weiss, 2009; McCabe et al., 2015). During the courageous actions, the anterior cingulate cortex (ACC) was activated whereas the amygdala was inhibited (Nili et al., 2010), which is implicated in processing fear and behavioral avoidance through the generation of autonomic responses to attenuate skin conductance and heart rate. In accordance with studies, the BIS was negatively associated with the ACC activity during rewarded action (Le et al., 2020; Sambuco et al., 2020). It might mean that courage was associated with larger suppression of BIS that positively activated the ACC region, therefore, the process of courageous action would have an inhibition effect on BIS motivational systems. It suggested a mechanism by which courage necessitated increased effort in order to overcome fear and buffer stress through inhibition of BIS in ACC activity was implicated in regulation of the autonomic nervous system (Nili et al., 2010) and cognitive control (Yee et al., 2022).

For BAS, only BAS-fun seeking was associated with stress and mediated the relationship of courage on stress, which could not verify the hypothesis. In terms of high risk occupations, employees must endure threat-adverse emotional response within negative emotions (McGurk and Castro, 2009). In order to be confronted with these dominating emotions evoked by threat, individuals need to evoke reversely fun seeking to decrease stress (Carver, 2004). As for BAS-reward responsiveness and drive, no significant correlations with stress were revealed since studies confirmed that anxiety might merely relate to hypersensitive BIS, as physiological mechanism controls the experience of anxiety in response to anxiety-relevant cues, but in contrast with a trivial relationship with BAS (Benjamin et al., 2020). Specifically, it theoretically stipulated that BAS was sensitive to reward. In those high-risk surroundings, the cost/benefit calculation would just inhibited the BIS activation, which was not enough to buffer stress. It was consistent with previous studies that individuals with PTSD would sacrifice reward to avoid threat (Weaver et al., 2020). Notwithstanding, results depicted that courage was positively associated with reward responsiveness and drive. The implications might prove that predispositional courage had the plausible inner psychological strength to drive employees to attain the noble goals, but the buffering effect on stress needed to be revealed (Taubitz et al., 2015).

## Limitation and further directions

The current findings should be evaluated in light of certain qualifications. Firstly, the effect of present study was cross-sectional. Further studies may utilize the longitudinal method to replicate the findings. Secondly, it is worth noting that the participants were collected in a sample of male firefighters. Future studies that enlarge sample size will be better able to explore variously for high-risk occupations. Thirdly, the mediating mechanism was assessed from the angles of statistics with self-report questionnaires among non-diagnosed populations. Future studies may design experimental paradigms on BIS/BAS for precisely testing the causal relationship between the courage and stress, and may reveal how neural mechanisms of behavioral control regulate from courage to stress (e.g., utilize functional magnetic resonance imaging technology to explore neural circuitry in the brain).

In high-risk settings, employees underwent the high prevalence of stress related disorders or symptoms that hinder individuals’ health and organizational effectiveness. Therefore, developing validated measures for the early detection and prevention is imperative. From the view of psychological evaluation, high-brave employees are more likely to be well-suited for high-risk occupations to buffer stress. Assessments need to identify such individuals for person-post matching. Crucially, behavioral inhibition and fun-seeking play an alleviating role between the courage and stress. Thus, the psychological training (e.g., fear control techniques) (Gondra et al., 2009), relaxation methods (Weisman and Rodebaugh, 2018), exposure therapy (Foa and Kozak, 1986) should be implemented to intervene, slowing down the inhibition system load and reducing the unpleasant feelings.

## Conclusion

The current study found the mediating effect of behavioral inhibition and fun-seeking on the association between courage and stress. Moreover, it provided important implications that might refer to behavioral inhibition and fun-seeking in stressful defense against high-risk environments, revealing the interplay of mediating processes in high-risk occupations and the protective factors of courage on employees.

## Data availability statement

The raw data supporting the conclusions of this article will be made available by the authors, without undue reservation.

## Ethics statement

The studies involving human participants were reviewed and approved by the Ethics Committee of Army Medical University (No. 2021-14-02). The patients/participants provided their written informed consent to participate in this study.

## Author contributions

JW and YW contributed to the design and conception of this study. JJ, HW, and XC participated in the statistical analysis. DS and QR participated in the experimental data collection. All authors actively took part in the process, read and approved the final manuscript.
